# Selenite Stimulates Mitochondrial Biogenesis Signaling and Enhances Mitochondrial Functional Performance in Murine Hippocampal Neuronal Cells

**DOI:** 10.1371/journal.pone.0047910

**Published:** 2012-10-22

**Authors:** Natalia Mendelev, Suresh L. Mehta, Haza Idris, Santosh Kumari, P. Andy Li

**Affiliations:** 1 Department of Pharmaceutical Sciences, Biomanufacturing Research Institute and Technology Enterprise (BRITE), North Carolina Central University, Durham, North Carolina, United States of America; 2 Department of Pathology, Ningxia Medical University, Yinchuan, Ningxia, People's Republic of China; Auburn University, United States of America

## Abstract

Supplementation of selenium has been shown to protect cells against free radical mediated cell damage. The objectives of this study are to examine whether supplementation of selenium stimulates mitochondrial biogenesis signaling pathways and whether selenium enhances mitochondrial functional performance. Murine hippocampal neuronal HT22 cells were treated with sodium selenite for 24 hours. Mitochondrial biogenesis markers, mitochondrial respiratory rate and activities of mitochondrial electron transport chain complexes were measured and compared to non-treated cells. The results revealed that treatment of selenium to the HT22 cells elevated the levels of nuclear mitochondrial biogenesis regulators PGC-1α and NRF1, as well as mitochondrial proteins cytochrome *c* and cytochrome *c* oxidase IV (COX IV). These effects are associated with phosphorylation of Akt and cAMP response element-binding (CREB). Supplementation of selenium significantly increased mitochondrial respiration and improved the activities of mitochondrial respiratory complexes. We conclude that selenium activates mitochondrial biogenesis signaling pathway and improves mitochondrial function. These effects may be associated with modulation of AKT-CREB pathway.

## Introduction

Selenium is a trace element necessary for normal cellular function in most animals including humans. Moderate selenium deficiency has been linked to several disorders including Keshan disease characterized by cardiomyopathy, Kashin-Beck disease characterized by osteoarthropathy, increased risk for certain cancers and infection, compromised immune response, hypothyroidism and neurodegerative disorders such as Alzheimer's disease [1]. Low dose supplementation of selenium (nanomolar range) has been shown to increase the levels of glutathione (GSH) and activities of glutathione peroxidases (GPXs), Thioredoxin reductases (TRXRs) and iodothyronine deiodinases [2]. Selenium protects cells from injuries induced by glutamate toxicity, oxidative stress, and inflammatory cytokines [3]–[8]. Selenium modulates several cell signaling pathways, including activation of the mitogen-activated protein kinase (MAPK), phosphotidylinositol 3-kinase (PI3K)-AKT, and NF-κB pathways [9], [10]. Although selenium is available in drug stores as a health supplement and its antioxidant effects have been proven *in vitro* and *in vivo*, its mechanism of action is not fully understood.

Mitochondria are both the powerhouse and source of ROS production in cells. Pathological conditions that cause increased free radical production instigate mitochondrial damage, resulting in release of proapoptotic factors that subsequently activate intrinsic apoptotic cell death pathways. Mitochondrial biogenesis, the process by which new mitochondria are formed, is activated in response to cellular stress. Although both the mitochondrial and nuclear genomes control the transcription and translation of mitochondrial proteins, the majority of mitochondrial proteins are transcribed by the nuclear genome. Peroxisome proliferator-activated receptor (PPAR) gamma coactivator-1 (PGC-1α and PGC-1β) and nuclear respiratory factors (NRF1 and NRF2) are master regulators of mitochondrial biogenesis [11]. PGC-1s are capable of binding to several nuclear receptors including NRFs, in addition to their interaction with PPAR. NRFs directly regulate the expression of several nuclear-encoded electron transport complex proteins, or indirectly regulate the three mitochondria-encoded cytochrome *c* oxidase (COX) subunit genes by activating mitochondrial transcription factor A (Tfam), which is responsible for the transcription of nuclear-encoded mitochondrial proteins. These proteins include both structural mitochondrial proteins and those involved in mitochondrial DNA (mtDNA) transcription, translation, and repair [12]–[16]. Selenium has been shown to activate phosphorylation of AKT, an upstream regulator of PGC-1α [10]. We hypothesize that selenium may stimulate the mitochondrial biogenesis signaling pathway and enhance mitochondrial functional performance. To test this hypothesis, we measured nuclear mitochondrial biogenesis regulating factors PGC-1α and NRF1, levels of mitochondrial proteins, and functions of mitochondria and activities of respiratory complexes in selenite- and non-selenite-treated mural hippocampal HT22 neuronal cells. To further delineate the upstream signaling pathways that are acted upon by selenium, we detected phosphorylation of AKT, CREB and PKA, and measured phospho-CREB and PGC-1α levels in the presence of selenium and inhibitors of Akt and PKA. Our results demonstrate that supplementation of selenium significantly increases the levels of mitochondrial biogenesis markers and mitochondrial protein levels, and improves mitochondrial functional performance and respiratory complex activities. Furthermore, selenium activates the mitochondrial biogenesis signaling pathway through phosphorylation of AKT.

## Results

### Selenium increases mitochondrial biogenesis markers and mitochondrial proteins

**Figure 1:**
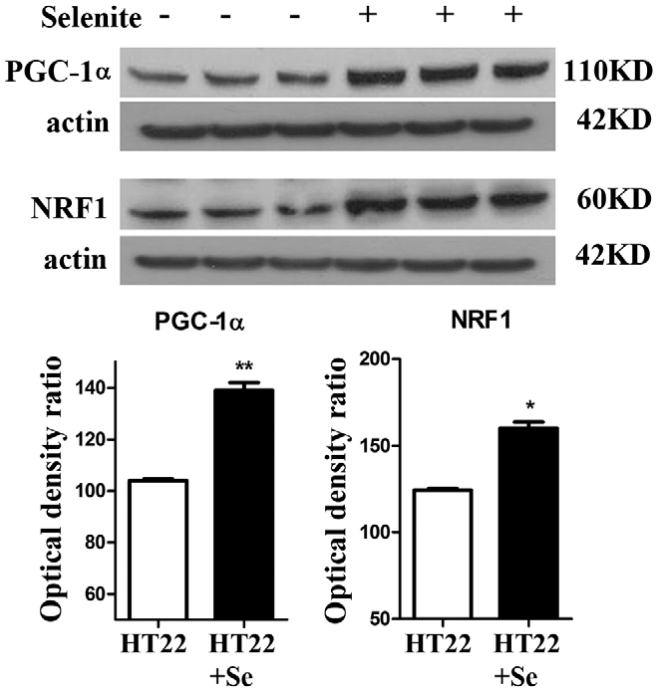
Supplementation of selenium enhances protein levels of mitochondrial biogenesis markers, PGC-1α and NRF1, in the nuclear fractions. *Upper panel*, representative Western blot bands. *Lower panel*, summarized bar graphs show band intensity presented as ratio of targeting protein over actin. Values are means±s.d. * p<0.05 and **p<0.01 by *t*-test.

**Figure 2:**
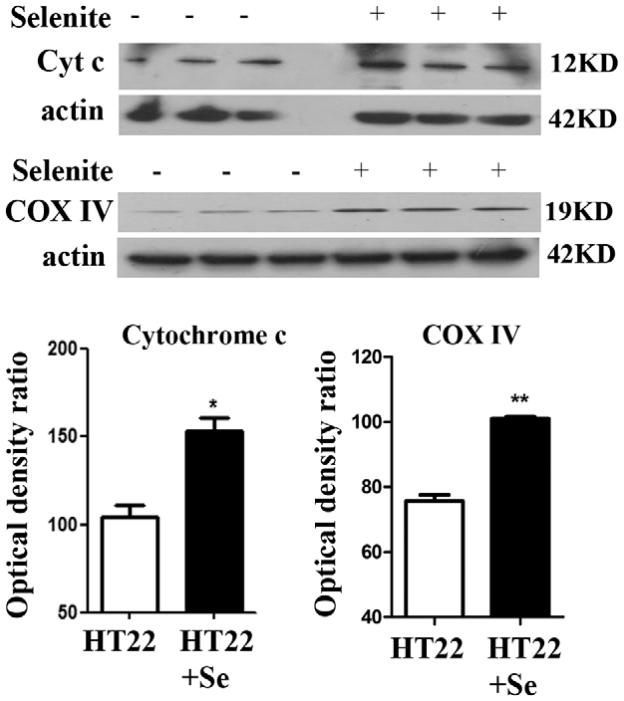
Selenite increases mitochondrial proteins. **A**, Western blotting bands of Cytochrome *c* and COX IV in the mitochondrial fractions. **B**, Summarized bar graphs shows band intensity presented as ratio of targeting protein over actin. Values are means±s.d. * p<0.05 and **p<0.01 by *t*-test.

The two key nuclear transcriptional factors, PGC-1α and NRF1, were used to evaluate the effects of selenium on mitochondrial biogenesis. As shown in **Fig. 1**, treatment of HT22 cells with 100 nM selenite for 24 h resulted in a 50% increase of protein levels of PGC-1α and NRF1 in the nuclear fraction. To verify whether elevation of nuclear PGC-1α and NRF1 increases mitochondrial mass, we measured two mitochondrial proteins, cytochrome *c* and COX IV. As demonstrated in **Fig. 2**, selenite treatment increased both proteins in the mitochondrial fraction.

To determine whether the activation effect of selenium on mitochondrial biogenesis markers is related to reduced free radical production due to selenium treatment, we treated cells with a well-known free radical scavenger Trolox (50 µM, Santa Cruz) for 24 h and then measured PGC-1 α and NRF1 in the nuclear fraction of the cell lysate. The results showed that Trolox failed to increase these two mitochondrial biogenesis markers (data not shown).

### Se increases phosphorylation of Akt and CREB

**Figure 3:**
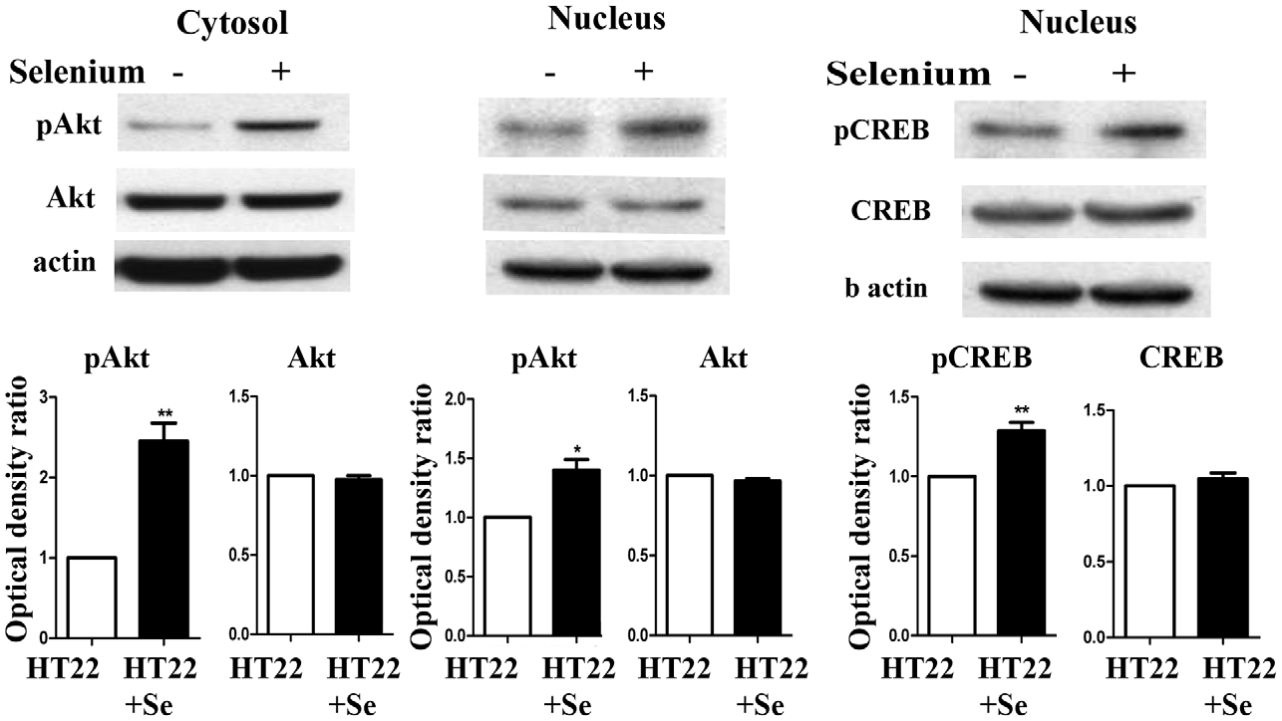
Selenite increases phospho-Akt in both the cytosolic and nuclear fractions and phospho-CREB in the nuclear fraction. *Upper panel*, representative Western blots for phospho-Akt (pAKT), total Akt, phospho-CREB (pCREB) and total CREB. *Lower panel*, summarized bar graphs presented as ratio of targeting protein over actin. Values are means±s.d. * p<0.05 and **p<0.01 by *t*-test.

PKA-CREB pathway has been shown to increase the transcription of PGC-1α (16). The transcription factor CREB can be phosphorylated at Ser 133 by Akt or PKA. To determine through which cell signaling pathway selenium promotes mitochondrial biogenesis signaling, we measured both phosphorylated and total protein levels of Akt CREB and PKA in both the cytosolic and nuclear fractions. The results demonstrated that treatment with 100 nM selenite significantly increased phospho-Akt in the cytosolic and nuclear fractions and phospho-CREB solely in the nuclear fraction without affecting the total Akt and CREB levels in the nuclear fractions (**Fig. 3**). However, neither phosphorylated nor total PKA protein levels were altered by selenium (data not shown).

**Figure 4:**
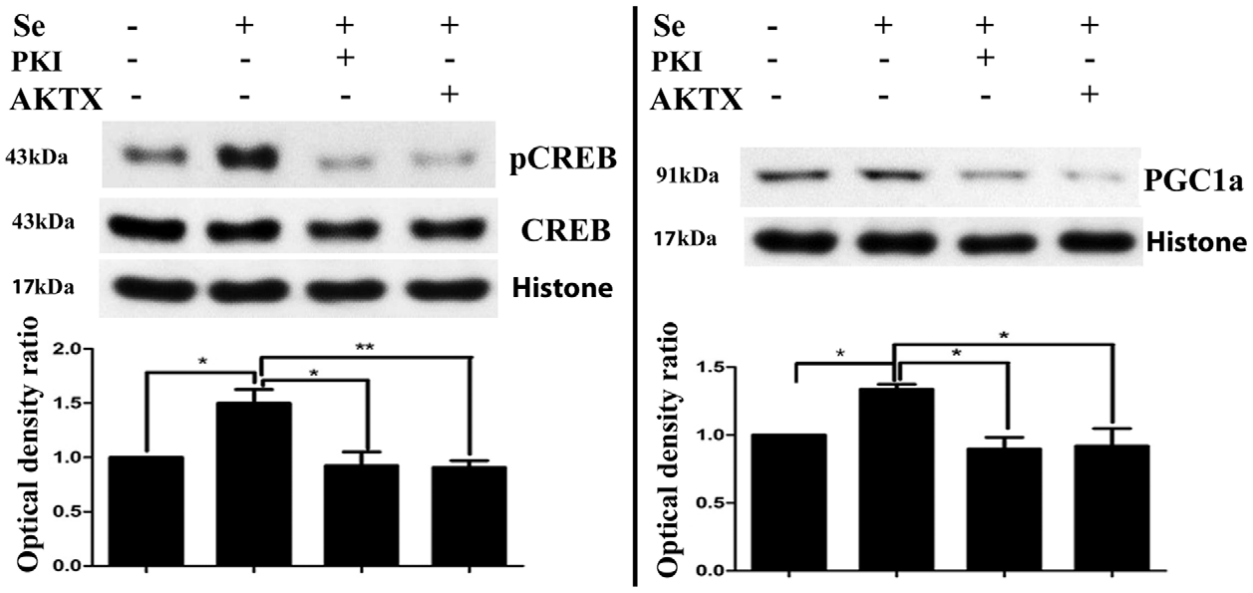
Effects of PKI and AKTX on Se-induced phospho-CREB and PGC-1α. Cells were treated with 100 nM Se. PKA inhibitor 14–22 amide or AKT inhibitor X (AKTX, 5 µM) were added to medium in the presence of Se. Western blotting was performed after 5 h of incubation. *Upper panel*, representative Western blot bands. *Lower panel*, summarized bar graphs show band intensity presented as ratio of targeting protein over Histone H3. Values are means±s.d. * p<0.05 and **p<0.01 by ANOVA followed by Tukey's test.

To further confirm whether selenium activates CREB and PGC-1α through Akt, we assessed the influence of selenium on CREB and PGC-1α in the presence of PKA inhibitor PKI and Akt inhibitor AKTX. As shown in **Fig. 4**, both PKI and AKTX blocked the selenium-induced phosphorylation of CREB and the increase of PGC-1α.

### Selenium improves mitochondrial respiration and complex activities

**Figure 5:**
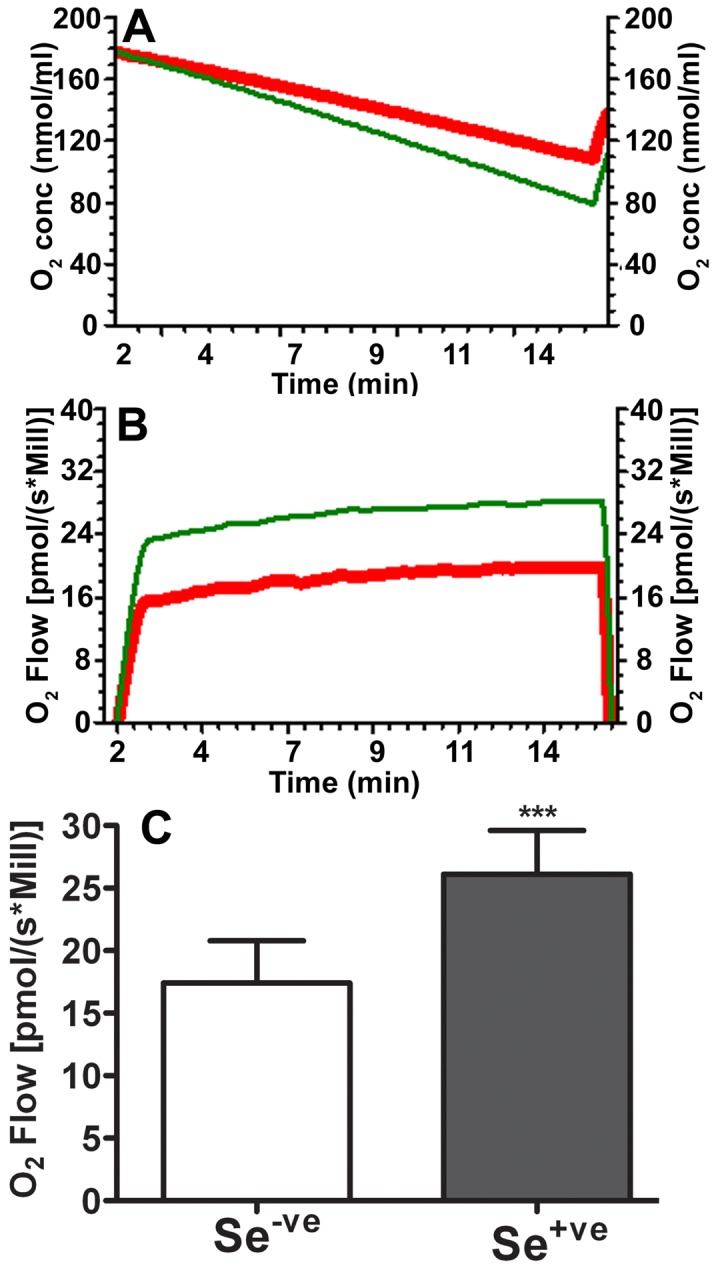
Selenite enhances mitochondrial respiration in HT22 cells. **A**, a typical recording of oxygen content reduction in non-selenium- (red curve) and selenium-treated (green curve) cells ; **B**, a respiration rate in non-treated (red) and selenium-treated (green) HT22 cells; ***C***, summarized mitochondrial respiratory rate obtained from 3 independent experiments in non-selenium treated (Se^−ve^) and selenium treated (Se^+ve^) cells. Values are means±s.d. *** p<0.001 by *t*-test.

To determine whether the stimulation of mitochondrial biogenesis by selenium leads to any functional gain of the mitochondria, we measured mitochondrial oxygen consumption and calculated mitochondrial respiratory rate. Our results showed that selenium treatment of HT22 cells resulted in increased oxygen consumption, thereby improving the mitochondrial respiratory rate compared with non-selenium treated cells. Respiratory rate was 17.4±3.4 in non-treated cells, while that increased significantly to 26.5±3.5 [pmol/(s*Mill)] in selenite-treated cells (p<0.001). Thus, selenium treatment increased the respiration rate by ∼36% in HT22 cells (**Fig. 5**).

**Figure 6:**
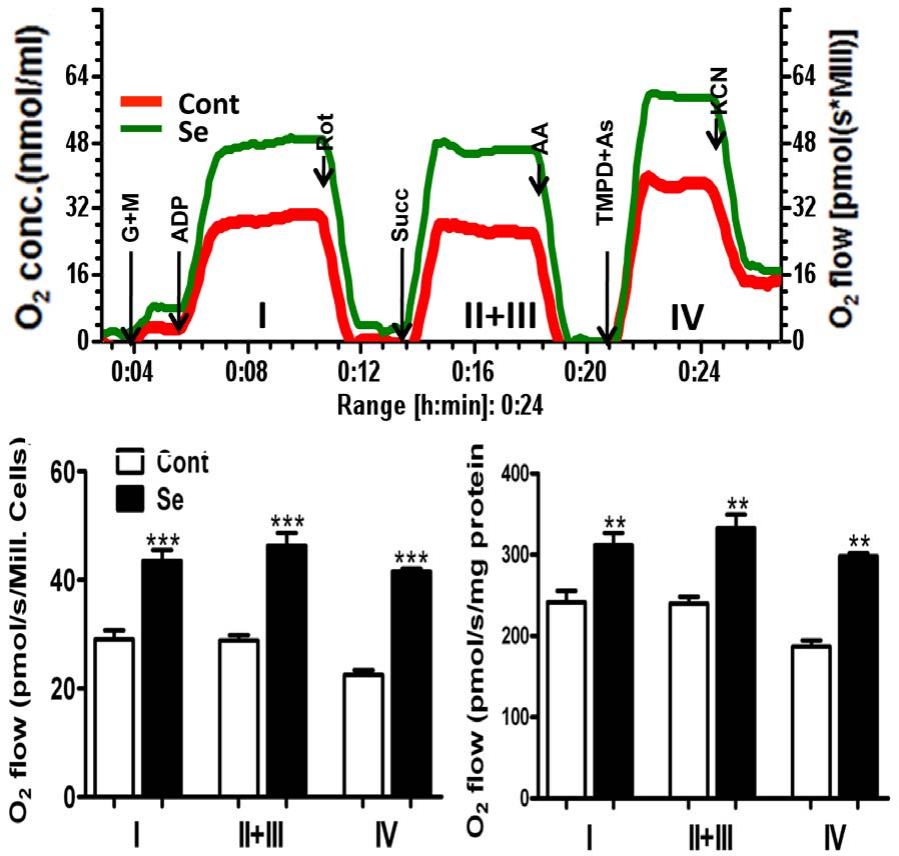
Selenite increases the activities of mitochondrial respiratory complexes I, II+III and IV. Oxygen consumption in digitonin permeabilized cells was measured using O2K oxygraph with addition of multiple substrates and inhibitors. The activities of each complex were calculated from the difference in reduction in oxygen content in the presence of specific substrates and inhibitors. Top panel is a polarograph recording showing mitochondrial respiratory activities of complex I, II+III and IV in control (red curve) and Se-treated (green curve) neurons. The mitochondrial enzyme activities were increased by selenite when counted both as oxygen flow rate per million cells (bar graph on left) and per milligram protein (bar graph on right). Values are means±s.d. **P<0.01, ***P<0.001 vs. non-se controls. AA, antimycin A; As, ascorbate; G+M, glutamate and malate; KCN, potassium cyanide; Rot, rotenone; Sc, succinate; TMPD, N,N,N',N'-tetramethyl-p-phenylenediamine dihydrochloride.

To further studied whether increased mitochondrial respiration is related to an increase in the activity of mitochondrial complexes, we measured oxygen utilization using complex specific substrates and calculated the activities of each mitochondrial respiratory complex from the difference in oxygen content reduction in the presence of specific respiratory complex inhibitors. As shown in **Fig. 6**, selenite treatment not only increased activity of complex I, II+III and IV in cells by 50, 60, and 85%, respectively, but also increased the activities per milligram of protein, suggesting that besides mitochondrial biogenesis, selenium may also increase the efficiency of each complex. To verify whether the observed selenium effect on the mitochondria is short-termed or long-lasting, we removed selenium from the culture medium after 24 h of incubation and then measured activity of mitochondrial respiratory complexes 6 h after the selenium being removed. The results showed that complex I and complex II and III activities after 6 h of selenium withdrawal was at the same level as those obtained right after 24 h of selenium treatment. Mitochondrial complex IV activity was slightly decreased compared with that without selenium removal, but the level remained significantly higher than in the non-selenium treated cells (data not shown).

## Discussion

Whether low dose of selenium supplementation is beneficial or harmful is still under debate. The beneficial effects of selenium on human health have been known for several decades. Selenium supplementation has been shown to prevent or completely reverse Keshan disease, to improve thyroid hormone metabolism by increasing deiodinase enzyme activity, to reduce the diarrheal morbidity in AIDS patients [18], to protect neurons against hypoxic/ischemic injuries [8], [19]–[23], and to inhibit growth of certain cancers (for reviews see [1], [24], [25]). It is known that high selenium dosage causes cytotoxicity. However, studies also showed that low dose selenium (0.1 µM) caused DNA fragmentation in primary cultured murine cortical neurons [26] and cell death that was believed due to increased ROS production by selenium [27]. Our results obtained from a parallel study do not support low dose selenium as toxic since we observed no influence on cell viability when selenium dosage is ranged from 30–200 mM [28]. It seems that selenium toxicity may be cell type specific. For example, while 100 nM selenite is toxic to human neuroblastoma SKNBE cells, it does not affect the cell viability in human embryonic kidney 293 cells (HEK-293) and the prostate cell line PNT-1 [27]. Using the same dosage of selenite, we have observed protective effects against glutamate-induced cytotoxicity in HT22 cells [28] and cerebral ischemia and reperfusion injury in mice [29]. Our results support the notion that low dose selenium supplementation is beneficial.

It is generally believed that selenium exerts most or all of its protective effects by incorporating into selenoproteins as the amino acid selenocysteine. Although the functions of many selenoproteins remain to be characterized, some of them, such as GPXs, TRXRs, and selenoprotein P regulate redox signaling [30]–[33]. The cell signaling pathways that are influenced by selenium, however, remain a subject of investigation. Recent studies have shown that selenium increases ERK1/2 phosphorylation [34], deactivates apoptosis signaling-regulating kinase 1 (ASK1) and induces phosphorylation of p38 and JNK MAPK [34]. In a recent study conducted using cybrid cells harboring NARP (Neuropathy, Ataxia and Retinitis Pigmentosa) mutation, Wojewoda and colleagues demonstrated that selenium supplementation increased levels of NRF1 and nuclear accumulation of NRF2, caused no change in levels of PGC-1α and Tfam, and significantly lowered phospho-Akt levels [35]. Our data show that selenite increases translation of PGC-1α and NRF1, two key regulators stimulating mitochondrial biogenesis. Subsequently, levels of two mitochondrial proteins, cytochrome *c* and COX IV, are increased in Se-treated cells comparing with non-treated controls, suggesting that increased mitochondrial biogenesis signal has led to enhancement of mitochondrial protein synthesis. Since selenium may exert its function through incorporation into selenoproteins, which possess antioxidant properties, we wanted to determine whether selenium's effects on PGC-1α and NRF1are due to reduction of free radicals by selenium. Therefore, we treated the cells with a well-know free radical scavenger Trolox for 24 h and then measured protein levels of PGC-1 α and NRF1. The results showed that neither PGC-1α nor NRF1 were increased by Trolox treatement, suggesting it is unlikely that the amount of free radical produced under physiological conditions influences the mitochondrial biogenesis.

Both PKA-CREB and AKT-CREB pathways can stimulate expression of PGC-1α and NRF1 [15]. Our results show that selenium fails to increase the phospho-PKA level in the nuclear fraction of the cell lysate after 24 h of incubation with selenium (data not shown). However, addition of the PKA inhibitor PKI blocked the phosphorylation of CREB and the increase of PGC-1α. These findings suggest that PKA is an upstream factor regulating CREB and PGC-1α; however, the effects of selenium do not seem to be mediated by PKA. Our study reveals that selenite increases phospho-Akt in both the cytosolic and nuclear fractions and phospho-CREB in the nuclear fraction. Akt phosporylates some thousands downstream cytoplamic and nuclear substrates, therefore it is not surprising that phospho-Akt can be found in both compartments [36]. Akt phosphorylation in the cytosol may causes translocation of phosphorylated Akt to other compartments [37]. To further confirm the role of Akt in mediating selenium effects on mitochondira, we applied Akt inhibitor AKTX in selenium-treated cells. The results show that inhibition of Akt blocked the effects of selenium on phospho-CREB and PGC-1α. These results suggest that the effect of selenium on PGC-1α is mediated through a Akt-CREB signaling pathway. Previous studies have demonstrated that selenite reduces free radical-induced apoptosis through activation of PI3-K/Akt pathway and inhibition of ASK1/JNK pathway [10], [38]. Since activation of Akt also regulates energy metabolism, maintains mitochondrial membrane potential and enhances adenosine triphosphate (ATP) production, this prompted us to investigate the influence of selenium on mitochondrial respiration and respiratory chain complex activities.

Our results show that selenite treatment significantly increases oxygen consumption, thereby improving mitochondrial respiration. This effect lasted at least 6 h after removal of selenium from the culture medium. Furthermore, selenite enhances the activities of mitochondrial respiratory chain complexes I, II+III and IV. As shown in **Fig. 6**, the mitochondrial enzyme activities were increased by selenium when counted both as oxygen flow rate per million cells (bar graph on left) and per milligram protein (bar graph on right), suggesting a real increase of mitochondrial complex enzyme activity. These results suggest that selenium enhances mitochondrial functional performance. Mitochondria play key roles in cell survival and death. Mitochondrial deterioration is a major pathophysiology of the aging process. Mitochondria initiate an intrinsic cell death pathway after being stressed. Therefore, preserving mitochondrial function by selenium may slow down the aging process and ameliorate cell death induced by various stress factors such as hypoxia, stroke, and inflammation. Indeed, published studies have reported that dietary selenium protects against selected signs of ageing [39], enhances ATP production in traumatic brain injury [40], increases the activities of mitochondrial respiratory chain complexes II, III, and IV [41] and inhibits mitochondria-initiated activation of the capase-9 and caspase-3 cell death pathway [10] after oxidative stress or hypoxic/ischemic injuries.

## Conclusion

Our data suggests that selenite can activate signaling pathways that stimulate mitochondrial biogenesis and increase mitochondrial protein synthesis. These effects are likely mediated through activation of the AKT-CREB pathway. In addition, selenite enhances mitochondrial respiration and complex activities.

## Materials and Methods

### Cell maintenance

The murine hippocampal HT22 cells, which were a generous gift from Dr. J. Panee (University of Hawaii) [17], were propagated in Dulbecco's Modified Eagle Medium (DMEM)/F12 (Invitrogen) containing 10% fetal bovine serum (FBS; HyClone SH30071.03), 2 mM glutamine, and 200 mM streptomycin/penicillin (Invitrogen) and then maintained at 90%–95% relative humidity in 5% CO_2_ at 37°C. The culture medium was renewed every 3 days. All experiments were performed in triplicate with at least 2 repetitions.

### Selenium dosage selection

In our pre-experiment, HT22 neuronal cells were incubated with sodium selenite (Na_2_SeO_3_; Sigma, cat. 214485) at the concentrations of 30, 50, 100, 200, and 500 nM. Cells were harvested after 24 h of incubation and cell viability was assessed using the 3-(4,5-dimethylthiazol-2-yl)-2,5-diphenyltetrazolium bromide (MTT) assay. The results showed that selenite up to the 200 nM dose range was well tolerated by HT22 cells without any influence on cell viability, whereas higher amounts of selenium (500 nM) led to 50% cell death after 24 h incubation. Based on this result and published literature, 100 nM selenium concentration, which is considered within physiological range [2], was used for subsequent studies.

### Treatment with Protein Kinase A inhibitor and Akt inhibitor

After reaching 85% confluence, cell were washed once with serum free DMEM medium and starved for 1 h. Protein kinase A inhibitor 14–22 amide (PKI 14–22, 5 µM, MERCK) or Akt inhibitor X (AKTX, 5 µM, Calbiochem) was applied in serum free media for 30 min. After 30 min, FBS was added to the culture to make a final concentration of 2% FBS with or without supplementation of 100 nM selenite. Cells were incubated for 5 h and the protein lysates were analyzed by Western blot. A concentration of 100 nM selenite was selected based on the results obtained from cytotoxicity assays.

### Western blot analysis

After 24 hours of sodium selenite treatment, cells were collected and lysed on ice in lysis buffer containing 20 mM Tris pH 7.4, 10 mM KCL, 3 mM MgCl_2_, 0.5% NP40, protease inhibitor cocktail (Roche) and phosphatase inhibitor cocktail (Halt Phosphatase Inhibitor Cocktail, Thermo Scientific). Lysates were centrifuged at 500×*g* for 10 min, resulting supernatant cytosolic fraction (S1) and pellet (P1). The P1 pellet was washed twice with lysis buffer, resuspended in lysate buffer containing 1%SDS and sonicated briefly on ice (Misonix, Ultrasonic Cell Disrupter). It was then centrifuged at 20,800×*g* for 30 min. The supernatants were designated as nuclear fractions. Protein lysates (25 µg) were separated in 4%–12% NuPAGE BT gels (Invitrogen), transferred to PVDF membrane (Millipore) and probed with the following antibodies: NRF1 1∶300, Santa Cruz), PGC-1α (1∶1000, Cell Signaling), Akt (1∶1000, Cell Signalling), phospho-Akt (1∶1000, Cell Signaling), CREB (1∶1000, Cell Signaling), phospho-CREB (1∶1000, Millipore), PKA(1∶1000, Cell Signaling), phospho-PKA (1∶1000, Cell Signaling), cytochrome *c* (Chemicon: 1∶1000 dilution) and COX IV (1∶1000, Abcam). Twenty micrograms of protein was loaded in each well and results were presented as band intensity ratio between target proteins and their loading controls.

### Measurements of mitochondrial respiration and complex activities

O_2_ consumption was measured using a respirometer (Oxygraph, Oroboros Instrument) equipped with a Peltier thermostat and electromagnetic stirrer. The measurement was done in a glass chamber containing 2 ml DMEM/F12 medium with10% FBS at 37°C. The medium containing 4.3×10^6^ cells was equilibrated in ambient room air with continuous stirring (750 rpm) for 10 min. The chamber was closed to start recording the oxygen consumption at 2 second intervals and the recording was stopped after stabilization of the O_2_ consumption. The difference in oxygen consumption between HT22 and HT22 with selenite treatment was calculated using DataGraph software (Oroboros Instruments) and represented as the basal respiration.

Respiration measurement at different complexes was performed polarographically to analyze activity of each complex using a multiple substrate-inhibition protocol (Chen et al., 2006). The experiment was conducted in the presence of 0.5 M ADP with a high resolution respirometer (Oxygraph, Oroboros Instrument) equipped with a peltier thermostat and electromagnetic stirrer at 37°C. Briefly, digitonin-permeabilized normal and selenium pretreated HT22 cells (1×10^7^) were incubated in 2 ml mitochondrial respiration medium MiR05 (110 mM sucrose, 0.5 mM EGTA, 3.0 mM MgCl_2_, 60 mM K-lactobionate, 10 mM KH_2_PO_4_, 20 mM Taurine, 20 mM HEPES, 1.0 g/l BSA, pH 7.1) in a glass chamber. The following substrates and inhibitors were used for complex I: glutamate (10 mM), malate (5 mM), and rotenone (0.5 µM); for complex II+III: succinate (10 mM) and antimycin A (2.5 µM) ; and for complex IV: N,N,N',N'-tetramethyl-p-phenylenediamine dihydrochloride (TMPD, 0.5 mM), ascorbate (2 mM) and KCN (1.0 mM). TMPD is frequently used in respiratory assays for cytochrome *c* oxidase activity as an artificial donor. In the assay, ascorbate is added as a regenerating system for reduced TMPD. To distinguish cellular respiration from the auto-oxidation of TMPD+ascorbate+cytochrome C complex IV, respiration is inhibited with potassium cynide (KCN). The pH of KCN was also neutralized to avoid TMPD auto-oxidation due to increase in pH. The integrity of the outer mitochondrial membrane following digitonin permeabilization was confirmed with cytochrome C (10 µM). The activity of each complex is represented as pmol/s/Million cells and as pmol/s/mg protein as well.

### Statistical analysis

All data were presented as means ± SD. Student's *t* test was used to analyze data presented in Figure 1, 2, 3, 5 and 6. ANOVA followed by post hoc Schffe's test was used to analyze data in Figure 4. A *p* value of less than 0.05 was considered significant.
